# One of the isoamylase isoforms, CMI294C, is required for semi-amylopectin synthesis in the rhodophyte *Cyanidioschyzon merolae*

**DOI:** 10.3389/fpls.2022.967165

**Published:** 2022-08-16

**Authors:** Toshiki Maeno, Yuki Yamakawa, Yohei Takiyasu, Hiroki Miyauchi, Yasunori Nakamura, Masami Ono, Noriaki Ozaki, Yoshinori Utsumi, Ugo Cenci, Christophe Colleoni, Steven Ball, Mikio Tsuzuki, Shoko Fujiwara

**Affiliations:** ^1^School of Life Sciences, Tokyo University of Pharmacy and Life Sciences, Hachioji, Japan; ^2^Faculty of Bioresource Sciences, Akita Prefectural University, Akita, Japan; ^3^Riken Center for Sustainable Resource Science, Yokohama, Japan; ^4^CNRS, UMR8576-UGSF-Unite de Glycobiologie Structurale et Fonctionnelle, University of Lille, Lille, France

**Keywords:** *Cyanidioschyzon*, floridean starch, isoamylase, red algae, semi-amylopectin

## Abstract

Most rhodophytes synthesize semi-amylopectin as a storage polysaccharide, whereas some species in the most primitive class (Cyanidiophyceae) make glycogen. To know the roles of isoamylases in semi-amylopectin synthesis, we investigated the effects of *isoamylase* gene (*CMI294C* and *CMS197C*)-deficiencies on semi-amylopectin molecular structure and starch granule morphology in *Cyanidioschyzon merolae* (Cyanidiophyceae). Semi-amylopectin content in a *CMS197C*-disruption mutant (*ΔCMS197C*) was not significantly different from that in the control strain, while that in a *CMI294C*-disruption mutant (*ΔCMI294C*) was much lower than those in the control strain, suggesting that CMI294C is essential for semi-amylopectin synthesis. Scanning electron microscopy showed that the *ΔCMI294C* strain contained smaller starch granules, while the *ΔCMS197C* strain had normal size, but donut-shaped granules, unlike those of the control strain. Although the chain length distribution of starch from the control strain displayed a semi-amylopectin pattern with a peak around degree of polymerization (DP) 11–13, differences in chain length profiles revealed that the *ΔCMS197C* strain has more short chains (DP of 3 and 4) than the control strain, while the *ΔCMI294C* strain has more long chains (DP ≥12). These findings suggest that CMI294C-type isoamylase, which can debranch a wide range of chains, probably plays an important role in semi-amylopectin synthesis unique in the Rhodophyta.

## Introduction

Most organisms store carbohydrates in the form of glucans. In photosynthetic organisms, cyanobacteria, Glaucophyta, Rhodophyta, Chlorophyta, Cryptophyta, and Apicomplexa contain α-glucans, while Stramenopiles, Haptophyta, Euglenophyta, and Chlorarachniophyta have β-glucans (Ball et al., 2015). In the α-glucan-containing organisms, most of cyanobacteria and only two genera of Rhodophyta (*Cyanidium* and *Galderia* in the most primitive class Cyanidiophyceae, subdivision Cyanidiophytina) synthesize soluble glucan, glycogen (Nakamura et al., 2005; Shimonaga et al., 2007, 2008), while other species produce starch granules. The starch of Chlorophyta, including green algae and plants, consists of amylopectin and amylose. On the other hand, starch-containing Rhodophyta have a different type of starch called floridean starch, which includes semi-amylopectin, an intermediate type of glucan between amylopectin and glycogen, in terms of the chain length distribution (Shimonaga et al., 2007, 2008; Hirabaru et al., 2010). The Rhodophyta are classified into the Cyanidiophytina, the Proteorhodophytina, and the Eurhodophytina (Guiry and Guiry, 2019). Species of the Proteorhodophytina accumulate glucan granules, including both semi-amylopectin- and amylose-type glucans (Shimonaga et al., 2007, 2008), while those of the Eurodophytina seem to have semi-amylopectin-type glucan alone-containing ones (Yu et al., 2002). In the most primitive subdivision Cyanidiophytina, *Cyanidium* and *Galdieria* have glycogen, as described above, while *Cyanidioschyzon* contains semi-amylopectin-type glucan alone, like the Eurodophytina (Shimonaga et al., 2007, 2008; Hirabaru et al., 2010). Although the cause of the structural difference between these species’ glucans has not been elucidated, differences in the properties and/or activities of enzymes functioning in glucan synthesis are expected to be involved.

As α-glucan synthesis-related enzymes, glycogen/starch synthase (GS/SS), branching enzyme (BE), and debranching enzyme (DBE), and glycogenin are known. GS/SS elongates α-1,4-glucosyl bond. BE cuts α-1,4-glucosyl bond and transfers the released glucan chain to form α-1,6-glucosyl bond, while DBE cleaves α-1,6-glucosyl bond. DBE is classified into isoamylase (ISA) and pullulanase (PUL) based on substrate specificity. ISA reacts with amylopectin and glycogen but cannot cut branches of pullulan, while PUL prefers pullulan and amylopectin to glycogen. Many isoforms of these enzymes with different properties have been found in Chlorophyta (for review, see Nakamura, 1996, 2015). Glycogenin is well known to be required for the initiation of glycogen synthesis in yeast and animals, and recently it has been demonstrated to be also involved in the floridean starch synthesis in *Cyanidioschyzon merolae* (Pancha et al., 2019a,b).

For ISA of land plants, there are three isoforms, ISA1, ISA2, and ISA3 (Nakamura, 2015). ISA1 and ISA2 function for starch synthesis as hetero-complex or homo-complex by trimming extra glucan chains to form the cluster structure of amylopectin, while ISA3 catalyzes starch degradation. ISA1-deficient *sugary* mutants accumulate phytoglycogen instead of starch in various plants, including maize, rice, and *Arabidopsis*. On the other hand, in Rhodophyta, analysis of ISAs has not been performed yet.

Rhodophyta have a unique glucan, semi-amylopectin (Yu et al., 2002; Shimonaga et al., 2007, 2008; Hirabaru et al., 2010). Recently, Pancha et al. revealed that the target of rapamycin plays the critical rules in floridean starch by changing the phosphorylation status of glycogenin in *C*. *merolae* (Pancha et al., 2019a,b). However, the enzymatic mechanisms underlying the unique structure of semi-amylopectin has not been fully clarified. To understand the mechanism, we investigated the roles of ISAs, using *ISA* gene-disruption mutants of *C. merolae*. The species has a genetic advantage in this purpose: a transformation system has been established, and homologous recombination tends to take place at a higher rate than non-homologous one, unlike other eukaryotic organisms (Minoda et al., 2004). Our study revealed that one of the ISAs, which cuts a wide range of α-1,4-glucan chains, plays an essential role in the semi-amylopectin synthesis. Also, the two mutants were demonstrated to have starch granules with unique morphologies.

## Materials and methods

### Algal cells and culture conditions

*Cyanidioschyzon merolae* cells were cultured in a modified Allen’s autotrophic medium (Minoda et al., 2004) under continuous illumination at 50 μmol photon m^−2^ s^−1^ with constant bubbling of air containing 2% CO_2_ at 42°C. A uracil-requiring strain M4 (Minoda et al., 2004), which was used for gene disruption, was grown in the presence of 0.5 mg ml^−1^ uracil, while its transformants with a marker gene *URA5.3* were cultured without uracil. Growth was monitored with OD_750_, cell number, and chlorophyll (Chl) *a*. The cell number was measured by Cellometer X2 Image Cytometer (Nexcelom Bioscience, Lawrence, MA, United States), while Chl *a* was extracted with 100% methanol, and its concentration was determined photometrically (Arnon et al., 1974).

### Construction of *CMI294C*- and *CMS197C*-disruptants of *Cyanidioschyzon*

A *CMI294C*-disruption mutant, *ΔCMI294C*, and a *CMS197C*-disruption mutant, *ΔCMS197C*, were constructed by introducing the respective DNA fragments for insertional mutagenesis to *C*. *merolae* M4 (Minoda et al., 2004) *via* homologous recombination as follows: DNA fragments containing *CMI294C*- and *CMS197C*-coding regions were amplified by PCR with primer sets *CMI294C*-F1 (5′-ACCGCCGAGTAAAGCATCTG-3′) and *CMI294C*-R1 (5′-TATCTTAGGGTGCCTGTTCG-3′) for the *CMI294C* region, and *CMS197C*-F1 (5′-TAACGGAGGAGCAAATGGAC-3′) and *CMS197C*-R1 (5′-TTCACAGCGAGGTTTCATCA-3′) for the *CMS197C* region, then both regions were cloned into the pGEM T-easy vector (Promega). The obtained plasmids carrying *CMI294C* and *CMS197C* DNA fragments were cut with *Bgl*II/*Nru*I and *Sfo*I (a blunt end-generating enzyme), respectively, and then ligated with 2.8 kb *URA5.3* DNA fragments. For *ΔCMI294C*, the *URA5.3* DNA fragment had been prepared by PCR with a primer set *Bgl*II-*URA5.3*-F (5′-ATAAGATCTGAACTGAGGGGCGAACGCA-3′) and *Nru*I- *URA5.3*-R (5′-ATATCGCGACCCTAGCAGCTGACTGTATC-3′) and then cleavage with *Bgl*II and *Nru*I. For *ΔCMS197C*, the *URA5.3* DNA fragment amplified with a primer set *URA5.3*-F (5′-TATTGATCAGAACTGAGGGGCG-3′) and *URA5.3*-R (5′-TGATGATCACCCTAGCAGCTGA-3′) was used for ligation, without restriction enzyme digestion. After transforming *C*. *merolae* M4 (Minoda et al., 2004; Imamura et al., 2010) with the disrupted gene DNA-fragments, transformants were isolated on plates not-including uracil. The obtained transformants *ΔCMI294C* and *ΔCMS197C* were confirmed by genomic PCR, using primer sets *CMI294C*-F2 (5′-GAAGAACCCTTCCACTGGGG-3′) and *CMI294C*-R2 (5′-AAGAGTTGCAAGCGAACGTG-3′), and *CMS197C*-F2 (5′-CGCCAGCTCGAGAACGCCTTAGC-3′), and *CMS197C*-R2 (5′-GGTGGGCGGTTGAAATCCGCACT-3′), respectively (Figure 2). As a control strain, a transformant with only a *URA5.3* gene was also generated.

### Measurement of the total α-glucan levels

Cells in about 10 ml of a culture were harvested by centrifugation (2,800 × *g*, 10 min, 4°C) and suspended in 100% ethanol. After vigorous vortexing, the suspension was centrifuged (15,000 × *g*, 10 min, 4°C), and then the pellet was dried up. The starch-containing pellet was solved in 1 ml of 0.2 M KOH by sonication, and then by boiling (for 30 min). The α-glucans were converted to glucose by treatment with glucoamylase (Seikagakukogyo, Tokyo, Japan), and the total glucose content was determined using hexokinase (Oriental Yeast Co., Ltd., Tokyo, Japan) and glucose-6-phosphate dehydrogenase (Roche Diagnostics K.K., Mannheim, Germany) according to Bergmeyer et al. (1974). Specifically, the glucose concentration in the glucoamylase-treated suspension was determined as follows: The suspension was centrifuged (15,000 × *g*, 10 min, 4°C), and then 150 μl each of the supernatant was placed into two holes of a microtiter plate, including 100 μl of NADP^+^-containing buffer [150 mM HEBES-NaOH (pH 7.4), 1 mM MaSO_4_, 0.3 mM NADP^+^, 1 mM ATP-Na_2_] with and without the enzymes (0.1 μl each). After 30 min-incubation, the absorbance at 340 nm was measured, and the glucose concentration was determined from the difference of the absorbance between with and without the enzymes.

### Separation of soluble and insoluble α-glucans

Algal cells were disrupted by sonication with a Sonifier 250D (Emerson, Danbury, CT, United States). Sonication was performed at output power scale 2 for 1 min 3 times, for 2 ml of 5-times concentrated cell suspension. The cell extract was centrifuged at 3,000 × *g* for 15 min. The supernatant was further centrifuged at 10,000 × *g* for 15 min, followed by ultracentrifugation at 1,00,000 × *g* for 1 h. The α-glucans in the pellet and supernatant fractions were determined as described above.

### Starch purification

*C. merolae* cells were ruptured by passing them twice through a French Pressure Cell at 2,000 kg/m^2^. The cell extract was centrifuged at 10,000 × *g* for 20 min at 4°C, and then the pellet was resuspended in 10 mM Tris–HCl (pH 8.0), 10 mM EDTA. For α-glucan purification, the suspension was layered onto 80% Percoll (Amersham Biosciences; 1.2 ml of 80% Percoll per 0.3 ml of the suspension), followed by centrifugation at 10,000 × *g* for 20 min at 4°C. Then, the glucan pellet was washed with distilled water.

### Capillary electrophoresis of debranched α-glucans

Chain length distributions of insoluble α-glucans were analyzed as follows (Hirabaru et al., 2010; Nakamura et al., 2020): Aliquots (20 mg) of starch obtained as described above were each suspended in 5 ml of methanol in a boiling water bath for 10 min, followed by centrifugation at 2,500 × *g* for 10 min. Each pellet was washed twice with 5 ml of 90% (v/v) methanol, and then suspended in 300 μl of 0.25 M NaOH. To the suspension were added 9.6 μl of 100% acetic acid, 100 μl of 600 mM Na-acetate buffer (pH 4.4), 15 μl of 2% (w/v) NaN_3_ and 1,090 μl of distilled water. Glucans in the sample was debranched with 6 μl (354 units) of *Pseudomonas amyloderamosa* ISA (Seikagakukogyo, Tokyo, Japan) at 37°C for 24 h, followed by incubation in a boiling water bath for 20 min. The resulting solution was used as the ISA-treated sample. The ISA-treated glucan was deionized by incubation with an ion exchange resin [about 5 mg of AG 501-X8 (D) Resin, BIO-RAD] in a microtube for 2 h at room temperature. An appropriate aliquot containing approximately 5 nmol of reduced end was evaporated to dryness in a centrifugal vacuum evaporator. Fluorescence labeling and capillary electrophoresis were performed according to O’Shea and Morell (1996), and the protocols provided with an eCAP N-linked oligosaccharide profiling kit and capillary electrophoresis P/ACE MDQ Carbohydrate System (Beckman Coulter, Carlsbad, CA, United States).

### SEM observation of starch granules

Starch granules were put on double-sided conductive carbon adhesive tape, and then osmium evaporation was performed from the top side. The surface morphology of each sample was examined using a scanning electron microscope (JCM-5700; JEOL, Tokyo, Japan).

### Quantitative PCR

Total RNA was isolated from 20 to 25-ml aliquots of cultures, by phenol-chloroform extraction, and then precipitated with ethanol (Tabei et al., 2007). The quantity and purity of the RNA were determined by absorption measurements at 260 nm and 280 nm with a spectrophotometer. For qPCR analysis of the starch synthesis-related genes [genes for ISA (*CMI294C*, *CMS197C*), SS (*CMM317C*), BE (*CMH144C*), and PUL (*CMP300C*)], total RNA was treated with RNase-free DNaseI (Takara Bio, Japan), and then reverse-transcribed with ReverTra Ace (Toyobo, Japan) and random primers (Takara Bio). qPCR was performed using Rotor-Gene SYBR® Green PCR kit (Qiagen, Germany) by a Rotor-Gene Q Real-time PCR System (Qiagen). PCR thermal profiles were 40 cycles of 95°C for 5 s and 60°C for 10 s. The PCR primers were designed based on the sequences available in the *C. merolae* Genome Project.^1^ The sequences of forward and reverse PCR primer sets were as follows: for *CMI294C*, 5′-TCTCAGCTGGAACTGTGGTG-3′ and 5′-GAAAAAGTTGCGCTCCTGAC-3′; for *CMS197C*, 5′-CCATGAATCGTTATGCGATG-3′ and 5′-ATCAAGGATGACCTCGATGC-3′; for *CMM317C*, 5′-CATCGGCTATGTGCGTATTG-3′ and 5′-GCGAAGGTAGTTCAGGAGAG-3′; for *CMH144C*, 5′-ATGCACGATATCGACACCAA-3′ and 5′-GGGATGGAAATTGAAAACGA-3′; for *CMP300C*, 5′-GTGGACTCGTGCAGTTACGA-3′ and 5′-GATACATCGGCGAACTTGCT-3′. All results were normalized to the expression level of the housekeeping gene *EF-1a* as an internal control (Fujiwara et al., 2009; GenBank accession number, XM_005536106). qPCR was performed on three biological replicates.

### Preparation of phylogenetic tree of DBEs

Sequences from *C. merolae* were used to retrieve sequences using homology searches by BLAST against sequences of the non-redundant protein sequence database of the NCBI and sequences from other databases (MMETSP and data publicly available). We retrieved the top 2,000 homologs with an E-value cut-off lower 1e^− 10^ and aligned them using MAFFT (Katoh and Standley, 2013) with the quick alignments settings. Block selection was then performed using BMGE (Criscuolo and Gribaldo, 2010) with a block size of 4 and the BLOSUM30 similarity matrix. We generated preliminary trees using Fasttree (Price et al., 2010) and ‘dereplication’ was applied to robustly support monophyletic clades using TreeTrimmer (Maruyama et al., 2013) in order to reduce sequence redundancy. The final set of sequences was manually selected and focused around Stramenopila sequences. Finally, proteins were re-aligned with MUSCLE, block selection was carried out using BMGE with the same settings as above, and trees were generated with IQTREE (Nguyen et al., 2015) under the LG4X model and bootstrap support values were estimated from 100 replicates.

## Results and discussion

### Phylogeny of ISAs

In land plants, ISA1 and ISA2 function for starch synthesis by trimming extra glucan chains to form clustar structure of amylopectin, while ISA3 catalyzes degradation of starch (Nakamura, 2015).

**Figure 1:**
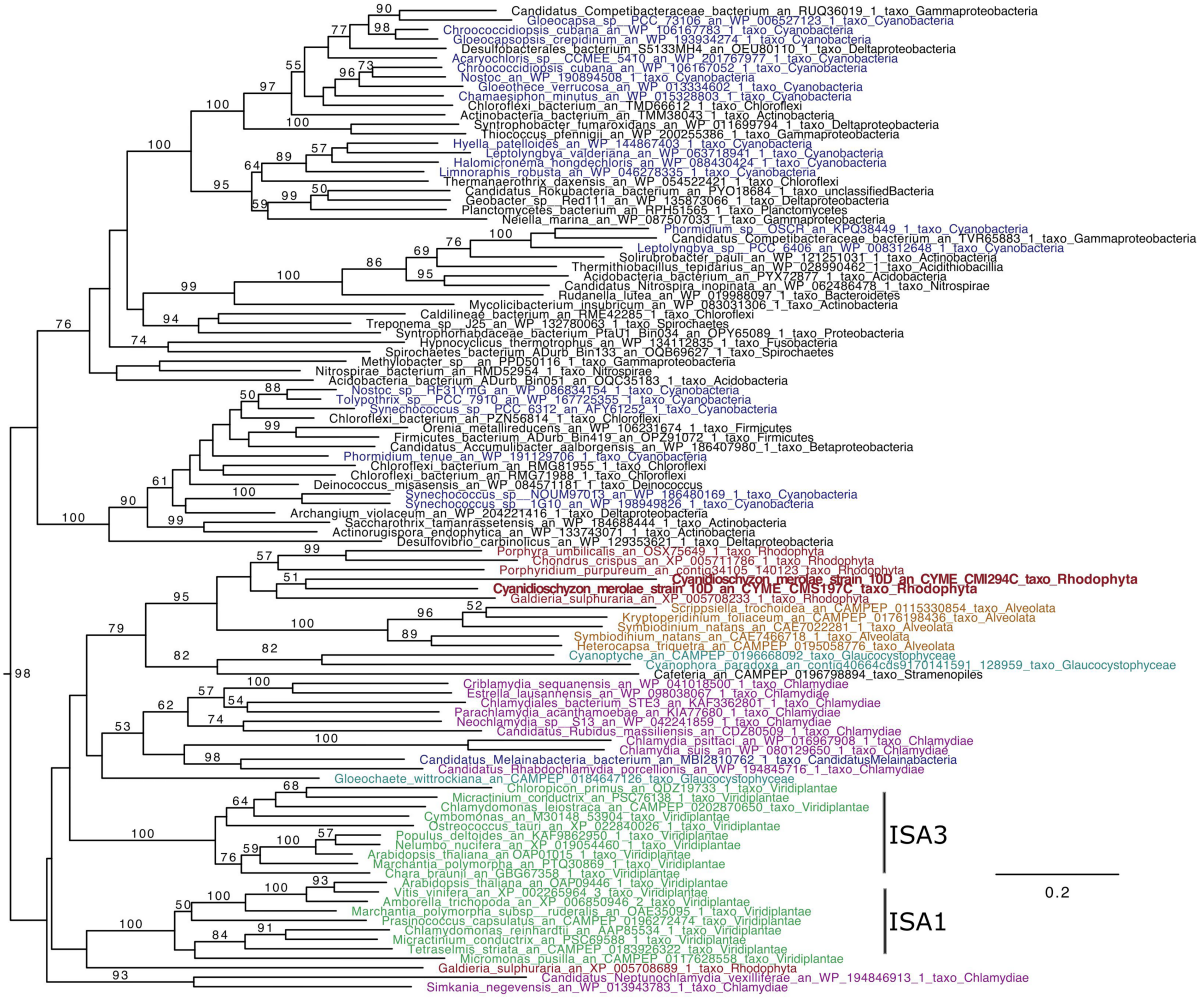
ISA phylogenetic tree (GH13_11). The tree was obtained using IQTREE with the LG4X model and bootstraps from 100 repetitions are mapped onto the branch, with only bootstrap values >50% shown. The scale bar shows the inferred number of amino acid substitutions per site, and the tree is midpoint rooted and visualized with Figtree. The Viridiplantae are in green, Rhodophyta in red, Chlamydiae in purple, Cyanobacteria in blue, Glaucocystophyceae in light blue, and Alveolata are in brown.

To know phylogenetic positions of ISAs of *C*. *merolae* and understand if it is possible to infer the functions, we performed phylogenetic analysis of ISAs (Figure 1). The ISAs that are present in plants and known to be involved in starch crystallization group with the ISAs from other Archaeplastida (Glaucocystophyceae and Rhodophyta) and eukaryotes which acquired plastids through secondary endosymbiosis, as well as Chlamydiae (BS = 98). Topology inside the Archaeplastida + Chlamydiae group is not well resolved. Nevertheless, the absence of ISA-like sequences in eukaryotes not affiliated with primary or secondary endosymbiosis suggests a chlamydial origin for the archaeplastidal enzymes. The presence of the candidate Melainabacterium sequence may be related to the parasitic way of life of some bacteria within this group notably toward green algae rather than its supposed common ancestry with cyanobacteria.

In Rhodophyta, *Galdieria*, *Porphyridium*, *Chondrus*, and *Porphyra* contain a single ISA gene, while *Cyanidioschyzon* displays two (*CMI294C* and *CMS197C*). The two *C. merolae* sequence group with other Rhodophyta. Thus, Rhodophyta ISAs are very interesting since they possess likely different characteristics, from plant ISAs as well as putatively between them, in terms of chain length preferences and glucan specificities of ISA1/2 (the universal chain-type), ISA3 (the very short chain-type), or cyanobacterial ISA (the intermediate chain-type; Kobayashi et al., 2016).

### Construction of a *CMI294C*- and a *CMS197C*-disruption mutants

**Figure 2:**
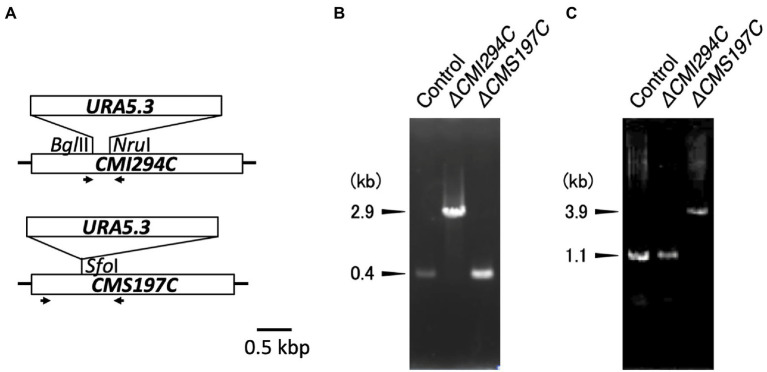
Disruption of the *ISA* genes in *Cyanidioschyzon*. **(A)** Strategy for disruption of the *ISA* genes through insertion of the *URA5.3* gene cassette. The *ISA* genes have simple structures without introns. Arrows indicate the PCR primers used for genomic PCR. **(B,C)** Confirmation of the *ISA* genes disruption by genomic PCR. PCR were performed with the primer sets *CMI294C*-F2 and *CMI294C*-R2 **(B)** and *CMS197C*-F2 and *CMS197C*-R2 **(C)**, using DNA of the control and the *ΔCMI294C* and *ΔCMS197C* strains of *C*. *merolae* as templates.

ISA gene (*CMI294C and CMS197C*)-disruption mutants of *C*. *merolae*, *ΔCMI294C* and *ΔCMS197C*, were generated by insertion of *URA5.3* (2.8 kb) into the respective target genes through homologous transformation. Each transformant was confirmed by genomic PCR, using a primer set that can amplify DNA region including the insert (Figure 2). For *ΔCMI294C*, the primer set, which amplifies a 0.4 kb DNA fragment of the wild type *CMI294C*, was used. As expected, a 0.4 kb band was observed using the control strain DNA as a template, while a 2.9 kb DNA band was detected with the *ΔCMI294C* DNA. For *ΔCMS197C*, the primer set which amplifies 1.1 kb DNA fragment including *CMS197C* with the control strain DNA as a template was used, and it was confirmed that a 3.9 kb DNA fragment, 2.8 kb longer due to the *URA5.3* insertion, was amplified with *ΔCMS197C* DNA. Thus, both kinds of ISA single mutants were successfully constracted, suggesting that the individual mutation is not leathal.

### Effects of the *CMI294C*- and the *CMS197C*-disruptions on growth and glucan content

**Figure 3:**
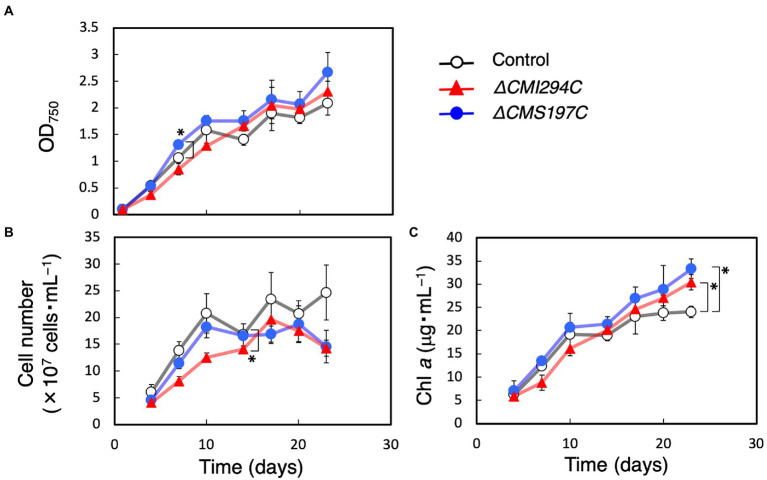
Effects of the *CMI294C*- and *CMS197C*-disruptions on the cellular growth. OD_750_ **(A)**, cell number **(B)**, and Chl *a* **(C)** of the control (◯) and the *ΔCMI294C* (▲) and *ΔCMS197C* (●) strains of *C*. *merolae* were measured. The bars represent means ± SD (*n* = 3; Student’s *t*-test, *p < 0.05, **p < 0.01).

**Figure 4:**
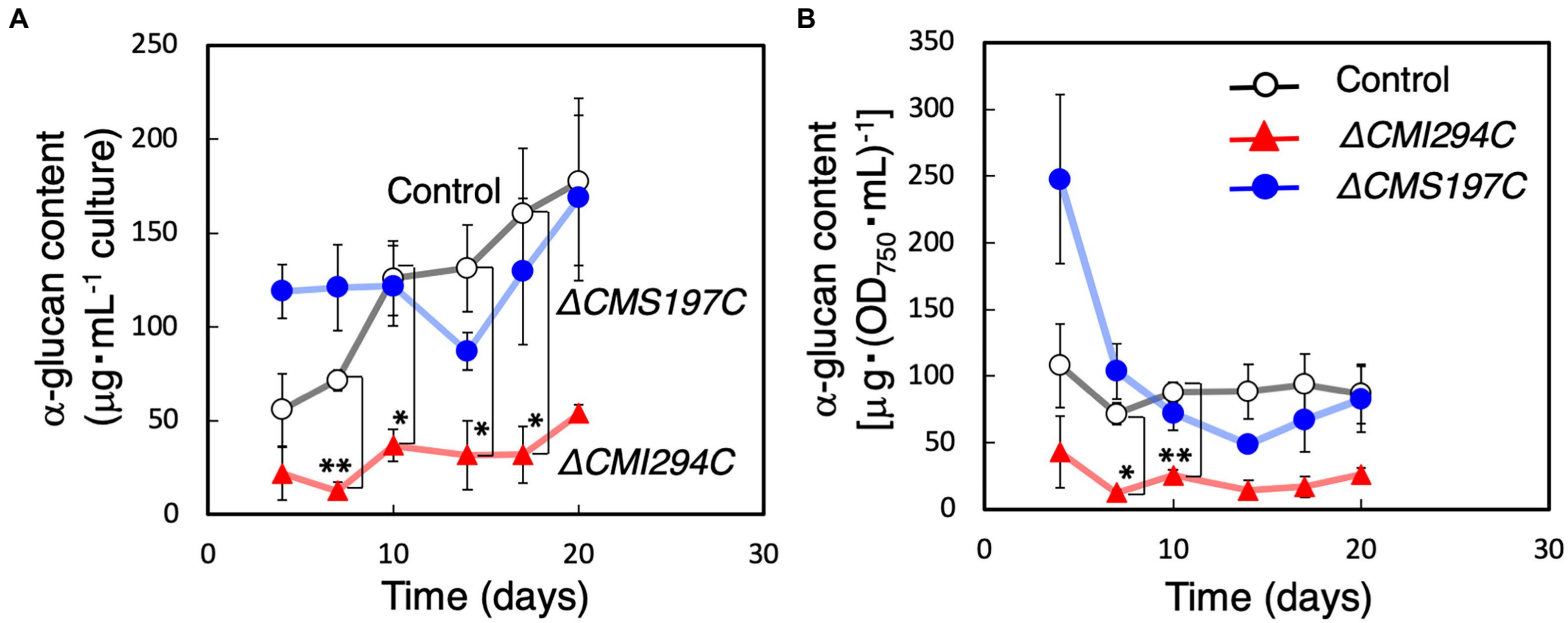
Effects of the *CMI294C*- and *CMS197C*-disruptions on total α-glucan content. The cellular total α-glucan content during the growth (Figure 3) was determined and plotted as α-glucan content per mL culture **(A)** or per cell included in 1 mL of OD_750_ = 1 culture **(B)**. ◯: control, ▲: *ΔCMI294C*, and ●: *ΔCMS197C*. The bars represent means ± SD (*n* = 3; Student’s *t*-test, *p < 0.05, **p < 0.01).

First, growth and total α-glucan content during growth were compared between the control and the obtained strains (Figures 3, 4). The time course of OD_750_, cell number, and Chl *a* were not significantly different between the control and the *ΔCMI294C* and *ΔCMS197C* strains, except for OD_750_ at 7 days, cell number at 14 days, and Chl *a* at 23 days (Figure 3). On the other hand, the ethanol-precipitated total α-glucan content in the *ΔCMI294C* strain was much less than in the control and the *ΔCMS197C* strains, although that in the *ΔCMS197C* strain was not so different from that in the control strain (Figure 4). This finding suggests that *CMI294C* is primarily involved in starch synthesis.

Since the total α-glucan content in the *ΔCMI294C* strain was much less than in the control strain (Figure 4), there was a possibility that photosynthetic product was accumulated as oligosaccharides instead of α-glucans in the *ΔCMI294C* strain. Thus, the content of saccharides in the water-soluble fraction, including oligosaccharides and water-soluble α-glucan, was investigated, and the rate in total saccharides was compared with those of the control and *ΔCMS197C* strains (Table 1). However, the rate of saccharides included in the water-soluble fraction was very low in the *ΔCMI294C* strain (1 ± 2%), and seemed to be hardly different from those of the control and *ΔCMS197C* strains. These findings suggest that in the *ΔCMI294C* strain photosynthesis may be repressed not to produce wasteful glucose units, or intermediate photosynthetic products may be converted to metabolites except for saccharides, such as lipids. Further analysis is necessary to clarify this point.

**Table 1:** Fractionation of α-glucans from *C. merolae* by centrifugation.

Fraction	Glucan yield (%)		
	Control	*ΔCMI294C*	*ΔCMS197C*
3,000 × *g* ppt	81 ± 6	79 ± 37	79 ± 28
10,000 × *g* ppt	13 ± 5	13 ± 12	16 ± 0
100,000 × *g* ppt	5 ± 1	7 ± 1	5 ± 1
100,000 × *g* ppt	1 ± 1	1 ± 2	0 ± 1

Extracts that were prepared by sonication were initially centrifuged at 3,000 × *g* for 15 min. The supernants were further centrifuged at 10,000 × *g* for 15 min, followed by ultracentrifugation at 10,000 × *g* for 1 h. Each fraction was converted to glucose by glucoamylase digestion. The total content of α-lucan in each fraction was determined using hexokinase and glucose-6-phosphate dehydrogenase according to Bergmeyer et al. (1974). Values are the means ± standard deviation for at least three replications.

### Gene expression of starch synthesis-related genes in the *ΔCMI294C* and *ΔCMS197C* strains

**Figure 5:**
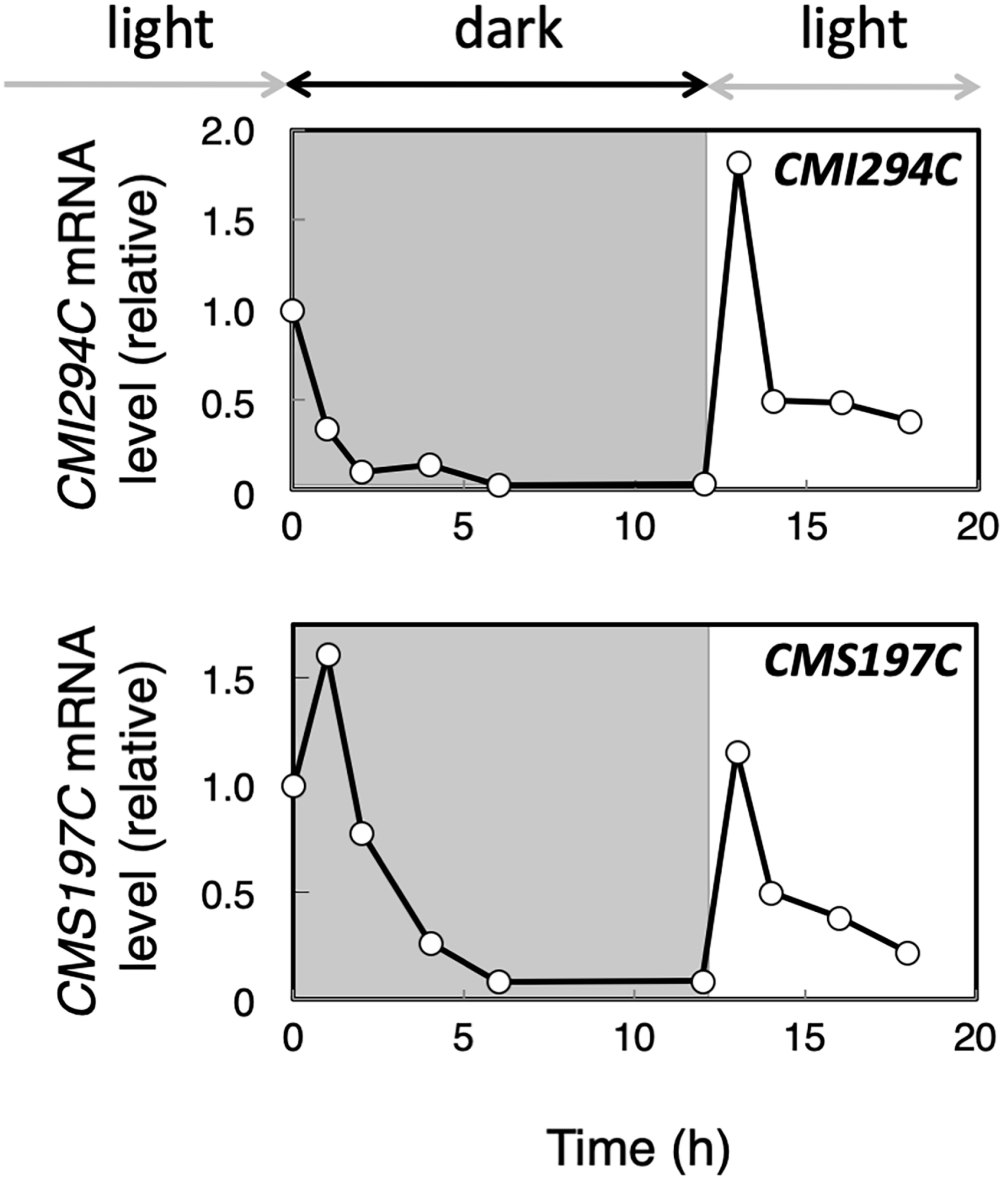
Expression of the *CMI294C* and *CMS197C* genes under dark and light conditions in the control strain. Cells grown in light were transferred to dark conditions for 12 h and then back to light conditions. The mRNA levels were measured by quantitative real-time PCR, using standard curves generated with determined numbers of the DNA fragments, and the mRNA levels of individual genes are plotted relative to those at 0 h. Identical trends were observed in another experiment (Supplementary Figure S1).

**Figure 6:**
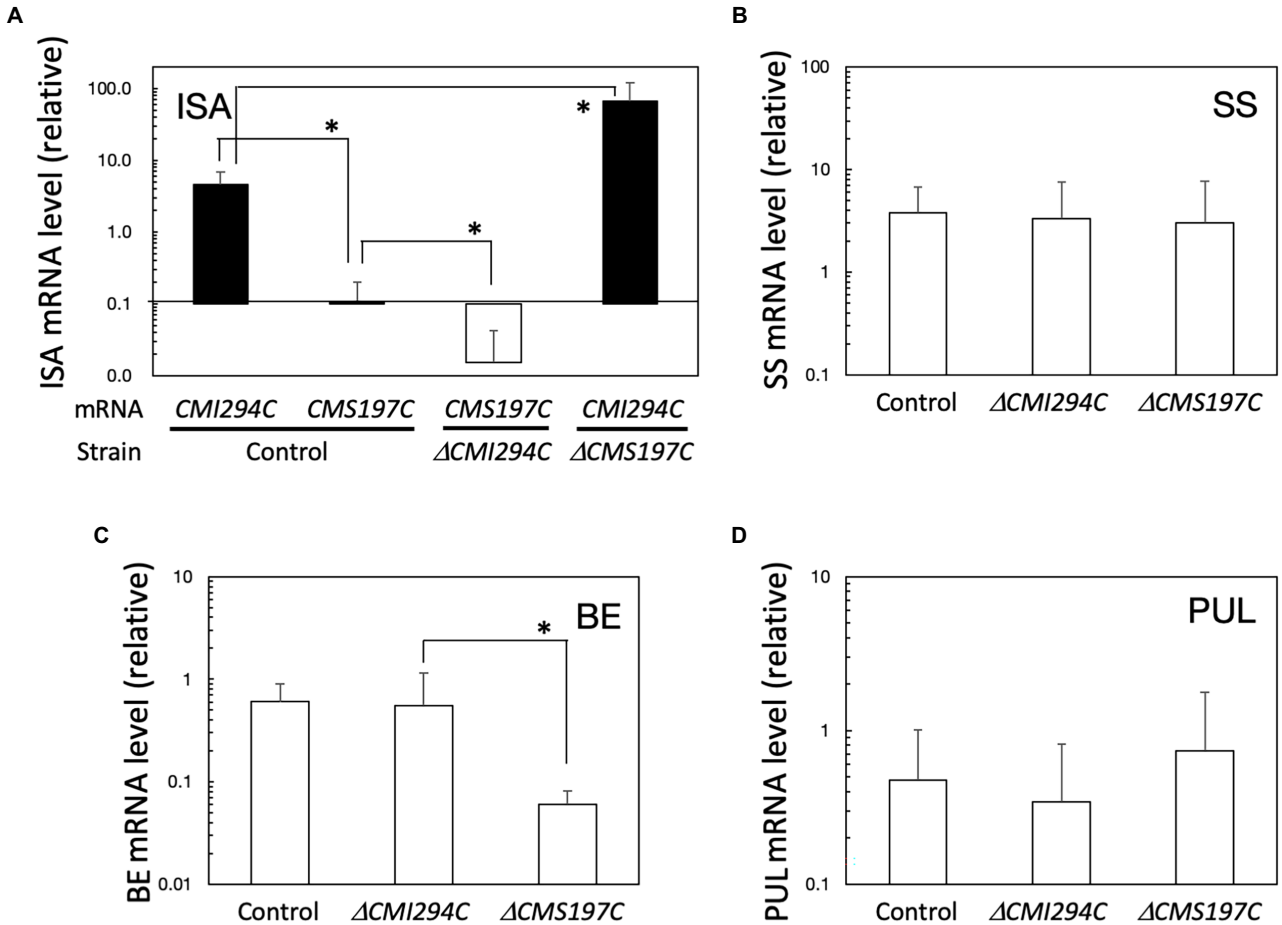
Expression of ISA **(A)**, SS **(B)**, BE **(C)**, and PUL **(D)** genes, in light in the control and the *ΔCMI294C* and *ΔCMS197C* strains. The mRNA levels were measured by quantitative real-time PCR, using standard curves generated with determined numbers of the DNA fragments. The mRNA levels of respective genes to the *EF-1a* mRNA level are plotted. The bars represent means ± SD (*n* = 3; Student’s *t*-test, *p < 0.05).

To infer whether *CMI294C* functions for starch synthesis but not for its degradation, we determined the effect of light on the gene expression in the control strain. Cultures were transferred to the dark and then moved back to light conditions, and then the time course of the *CMI294C* mRNA level was investigated, together with the *CMS197C* mRNA level (Figure 5). The level of *CMI294C* mRNA decreased in the dark and reached nearly 0 within 12 h. Then, after the transfer back to light, 1 h was enough to recover to a high expression level. A similar pattern was observed in the *CMS197C* mRNA level, although the mRNA level was much less than *CMI294C*. Under continuous light conditions, the *CMI294C* mRNA level/*EF-1a* mRNA level was 4.6 ± 2.4 (*n* = 3), while the *CMS197C* mRNA level/ *EF-1a* mRNA level was 0.11 ± 0.10 (*n* = 3; Figure 6A). It is speculated that both gene products function while photosynthesis is carried out and starch is actively synthesized, and that CMI294C is a predominant one.

The expression of these ISA genes under continuous light was also determined in the *ΔCMI294C* and *ΔCMS197C* mutants (Figure 6A). In the *ΔCMI294C* mutant, the *CMS197C* mRNA level relative to the housekeeping gene *EF-1a* mRNA level (Fujiwara et al., 2009) was significantly lower than in the control strain, while in the *ΔCMS197C* mutant, the *CMI294C* mRNA level to the *EF-1a* mRNA level was significantly higher than that in the control strain. It is speculated that CMI294C activity is normal in the *ΔCMS197C* mutant and thereby the strain can accumulate a normal level of starch in.

The expression of other starch synthesis-related genes, genes for SS, BE, and PUL, in light was also compared among the control and the mutant strains (Figures 6B–D). These genes were all expressed in light in the control strain, and the SS and PUL mRNA levels in *ΔCMI294C* and *ΔCMS197C* were not significantly different from those in the control strain, while the BE mRNA level in *ΔCMS197C* was significantly lower than the control and *ΔCMI294C* strains. It is speculated that the activities of the starch synthesis-related enzymes, except for the ISA (in both mutants) and BE (in *ΔCMS197C*) gene products, are probably not so different among these strains.

### Effect of the *CMI294C*- and the *CMS197C*-disruptions on chain length distribution of α-glucan

**Figure 7:**
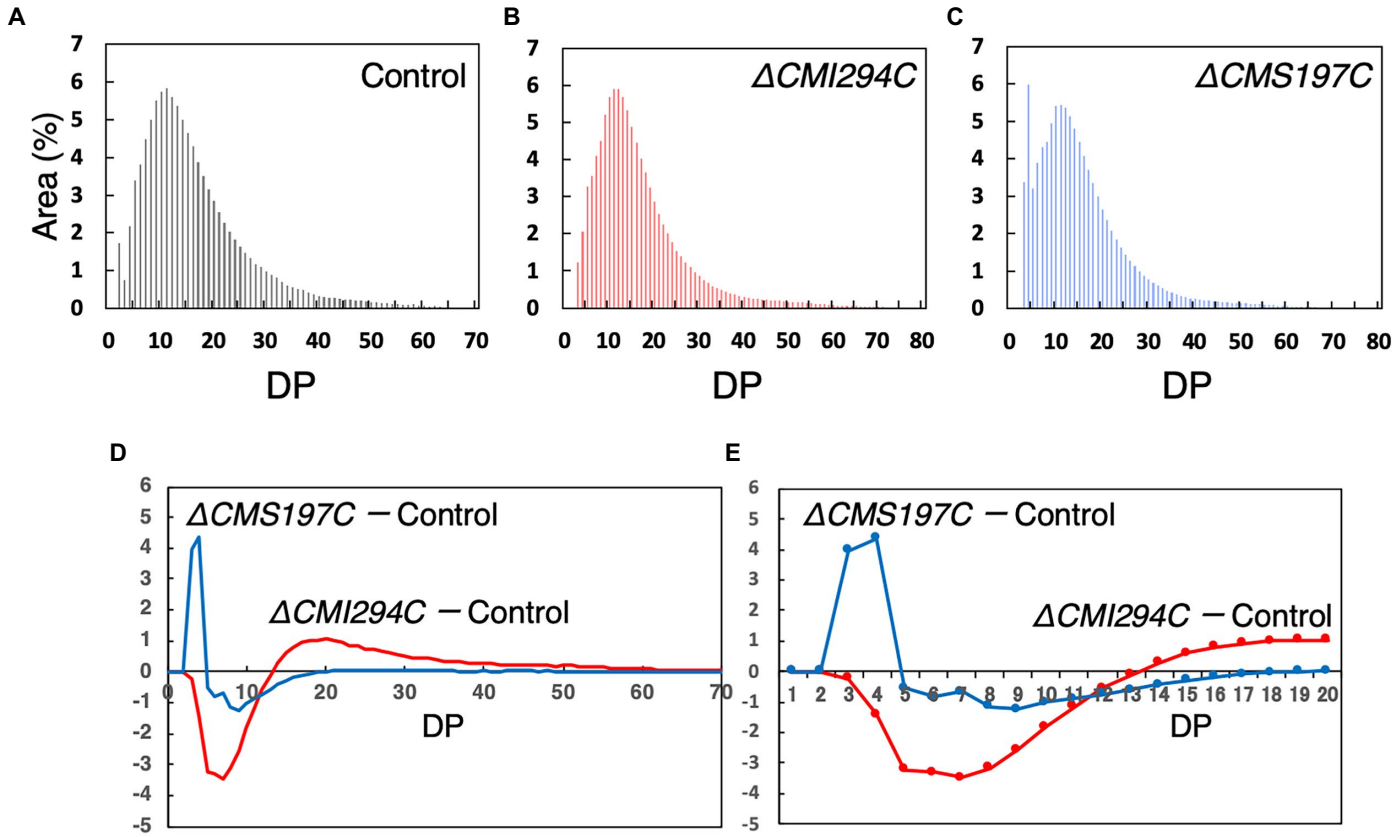
Comparison of the chain length distribution between starch from the control strain and those from the *ΔCMI294C* and *ΔCMS197C* strains. Starch samples were isolated from late-log phase cells grown under continuous illumination with constant air bubbling containing 2% CO_2_. The chain length profiles **(A–C)** and differences in chain length profiles **(D,E)** are shown. The abundance of chains with individual degrees of polymerization (DP) was plotted as area % of the chromatograms obtained by the capillary electrophoresis. **(E)** is an enlarged figure of **(D)** to clearly show DP numbers that are different between the strains. Identical trends were observed in another experiment (Supplementary Figure S2).

To infer the branch length specificities for CMI294C and CMS197C, we compared the chain length distribution of α-glucan between the control and the gene-disruption mutants (Figure 7). In the control strain, the chain length distribution displayed a semi-amylopectin pattern, as previously reported in the wild type: there was a peak around degree of polymerization (DP) 11–13, accompanied by a gradually declining curve without a peak around DP45, whereas the peak of long chains was essential for amylopectin, but not for semi-amylopectin (Figure 7A; Hirabaru et al., 2010). α-Glucan of *ΔCMI294C* had fewer short chains (DP ≤ 11) but more long chains (DP ≥ 12) than that of the control strain (Figures 7D,E). In contrast, the chain length distribution pattern in *ΔCMS197C* had a peak around DP4, in addition to a peak of DP11-13 (Figure 7C). Thus, α-glucan of *ΔCMS197C* had more short chains (DP3-4) and fewer long chains (DP5-19), when compared to the control strain (Figures 7D,E). These findings suggest that a minor ISA, CMS197C, is related to the removal of branches with very short chains (DP3-4), while a major ISA, CMI294C, is involved in the cleavage of a wide range of short and intermediate chains.

### Effect of the *CMI294C*- and the *CMS197C*-disruptions on glucan granule morphology

**Figure 8:**
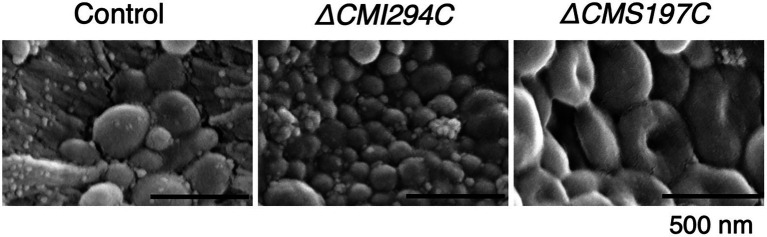
SEM images of glucan granules purified from the control and the *ΔCMI294C* and *ΔCMS197C* strains of *C*. *merolae*. Starch samples were isolated from late-log phase cells grown under continuous illumination with constant air bubbling containing 2% CO_2_. Scale bars, 500 nm.

Since the glucan content of the *ΔCMI294C* strain was significantly lower than the control strain, possibilities that the size or the number of glucan granule is decreased in the mutant were considered. To investigate these possibilities and the effect of chain length on the glucan morphology, we compared the morphology of isolated glucan granules by scanning electron microscopy (Figure 8). The glucan granules of the *ΔCMI294C* strain [average size of the granules excluding fine dot-like structures, 106 nm in diameter (*n* = 27)] were much smaller than those of the control strain [average size of the granules, 257 nm in diameter (*n* = 10)], although the morphology was not so different between the strains. On the other hand, the sizes of glucan granules of *ΔCMS197C* [average size of the granules, 375 nm in diameter (*n* = 10)] were almost the same as those of the control strain, but most of the glucan granules of the *ΔCMS197C* strain were donut-shaped, unlike those of the control strain having discoidal morphology without a hollow. These findings suggest that CMI294C positively impacts granule size and total glucan content, while CMS197C affects morphology to some extent.

### Hypothetical functions of CMI294C and CMS197C

**Figure 9:**
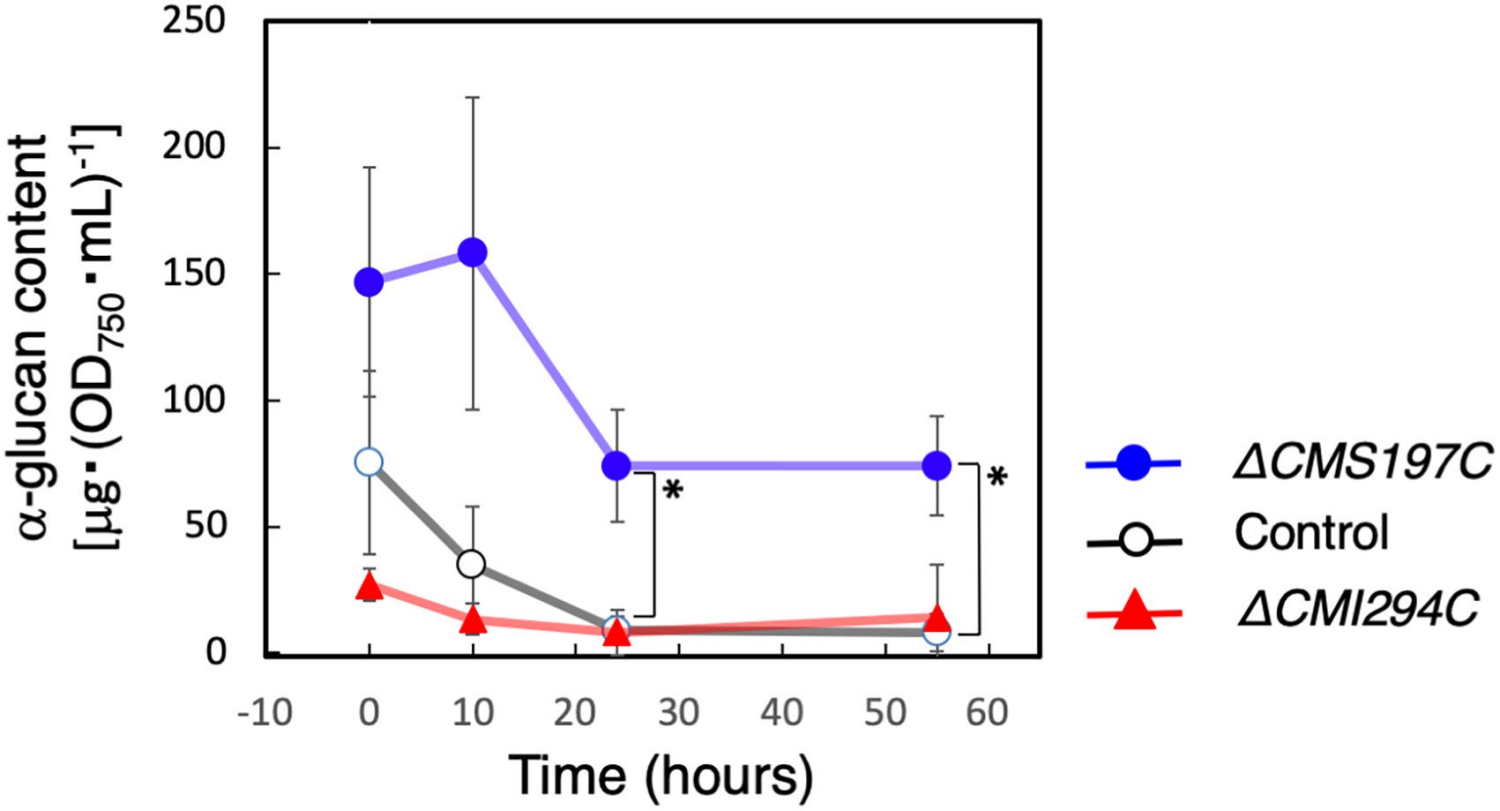
Effects of the *CMI294C*- and *CMS197C*-disruptions on α-glucan degradation in the dark. The cellular total α-glucan content during the incubation in the dark was determined and plotted as α-glucan content per cell included in 1 mL of OD_750_ = 1 culture. ◯: control, ▲: *ΔCMI294C*, and ●: *ΔCMS197C*. The bars represent means ± SD (*n* = 3; Student’s *t*-test, *p < 0.05).

Since CMS197C was suggested to almost exclusively debranch very short chains (DP3-4) as ISA3 of green plants (Figure 7; Kobayashi et al., 2016), it was also predicted to be involved in degradation of α-glucan, which side chains had been shortened by other enzymes such as amylases, phosphorylase, and/or disproportionating enzyme. To confirm this hypothesis, we compared the starch degradation rate in the dark between the *ΔCMS197C* and *ΔCMI2942C* strains and the control strain (Figure 9). In the *ΔCMI294C* strain and the control strain, starch was almost completely degraded within 24 h in the dark. In contrast, in *ΔCMS197C*, starch degradation was not observed so much until 10 h, and then the starch level was decreased gradually, but the degradation stopped around 24 h, suggesting that CMS197C is involved in starch degradation. Thus, CMS197C probably cleaves very short chains of DP 3 and 4 produced by actions of amylases, phosphorylase, and/or disproportionating enzymes, like ISA3 in green plants.

On the other hand, CMI294C was suggested to be involved in semi-amylopectin synthesis (Figure 4). The chain length preference seemed to be different from those in amylopectin-synthesizing green plants: rice recombinant ISA1 preferred chains of around 6 (Kobayashi et al., 2016), while CMI294C seemed to prefer chains of DP ≥ 14 (Figures 7D,E). This might account for the structural differences between amylopectin and semi-amylopectin, although characteristics and activities of other enzymes such as BE could be also important factors. The difference in chain length preference of ISA might account for more short chains of DP ≤ 8 in semi-amylopectin than in amylopectin, and a wider range of chain preference of CMI294C-type ISA in red algae could be related to no shoulder around DP45 in chain length distribution of semi-amylopectin (Figures 7D,E; Kobayashi et al., 2016; Hirabaru et al., 2010). To clarify this hypothesis, determination of chain length preference with recombinant enzymes, as reported by Kobayashi et al. (2016) are necessary, as well as analyses of other enzymes, including BE.

α-Glucans of other species of Cyanidiophyceae, *Cyanidium* and *Galdieria*, are glycogen which include about 90% of ≤DP10 chain (Shimonaga et al., 2008), while α-glucan of the *ΔCMI294C* mutant in *Cyanidioschyzon* has still different structure (semi-amylopectin). This means that CMI294C is important for semi-amylopectin synthesis in the rhodophytes, but it is not the only enzyme that is responsible for the synthesis of semi-amylopectin, but not glycogen. To know whether CMI294C-type ISA is responsible for the diversification of the α-glucans in the subdivision, BE of the subdivision, as well as ISA of *Cyanidium* and *Galdieria*, should be investigated. The difference in the mechanism of synthesis between glycogen and starch in Rhodophyta is still unclear. However, probably CMI294C-type ISA and combination with other enzymes are important for the development of semi-amylopectin granule.

In summary, the primitive rhodophyte *C*. *merolae*, which synthesizes semi-amylopectin alone, has two ISA genes in the genome. It was suggested that CMS197C cuts short branches of DP3-4, while a predominant gene product CMI294C cleaves a wide range of longer chains. Semi-amylopectin molecular structure, which is orderly trimmed by CMI294C, is probably essential for the development of glucan granules. These results obtained in the present study would provide useful knowledge for metabolic engineering to produce novel glucan materials with unique morphology or size, as well as interesting insight toward understanding of evolution process of glucan synthesis.

## Data availability statement

The datasets presented in this study can be found in online repositories. The names of the repository/repositories and accession number(s) can be found in the article/Supplementary material.

## Author contributions

TM, YY, YT, and SF planned and designed the research. TM, YY, YT, MO, and NO performed experiments and analyzed data. UC and YU conducted the preparation of phylogenetic tree. SF wrote the article with contributions from all authors. TM and YY contributed equally. All authors contributed to the article and approved the submitted version.

## Funding

This study received funding from JSPS KAKENHI Grant Number 20K06326, and the Promotion and Mutual Aid Corporation for Private Schools.

## Conflict of interest

The authors declare that the research was conducted in the absence of any commercial or financial relationships that could be construed as a potential conflict of interest.

## Publisher’s note

All claims expressed in this article are solely those of the authors and do not necessarily represent those of their affiliated organizations, or those of the publisher, the editors and the reviewers. Any product that may be evaluated in this article, or claim that may be made by its manufacturer, is not guaranteed or endorsed by the publisher.

## Footnotes

^1^http://merolae.biol.s.u-tokyo.ac.jp/ (Accessed July 25, 2022).
