# Supplementary material for: One of the isoamylase isoforms, CMI294C, is required for semi-amylopectin synthesis in the rhodophyte Cyanidioschyzon merolae
Source: Front Plant Sci. 2022 Aug 16;13:967165. doi: 10.3389/fpls.2022.967165 (PMC9424615; doi:10.3389/fpls.2022.967165)
Supplement: Supplementary file 1 [file Data_Sheet_1.PDF]

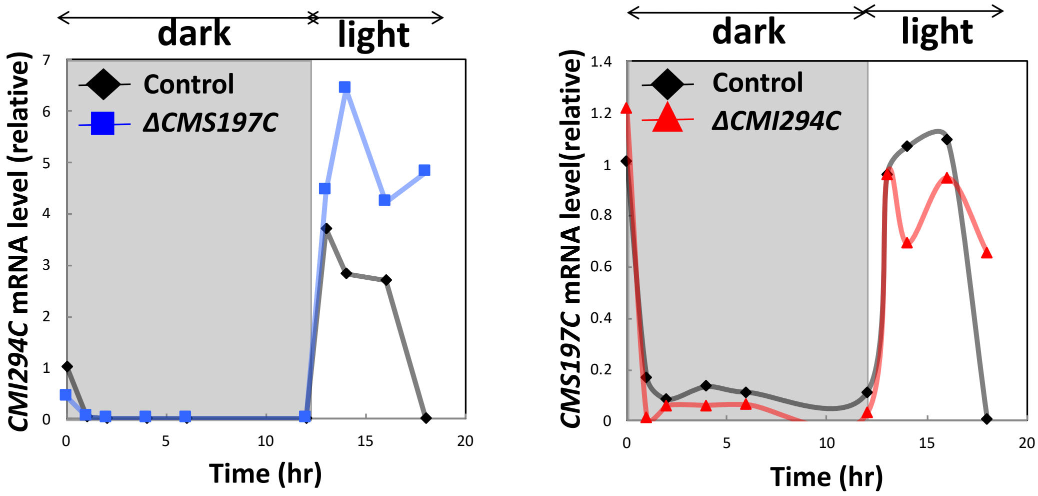

Fig. S1. Expression of the *CMI294C* and *CMS197C* genes under dark and light conditions in the control and the *ΔCMI294C* and *ΔCMS197C* strains. Cells grown in light were transferred to dark conditions for 12 h and then back to light conditions. The mRNA levels were measured by quantitative real-time PCR, and the mRNA levels of individual genes are plotted relative to those at 0 h.

a Control

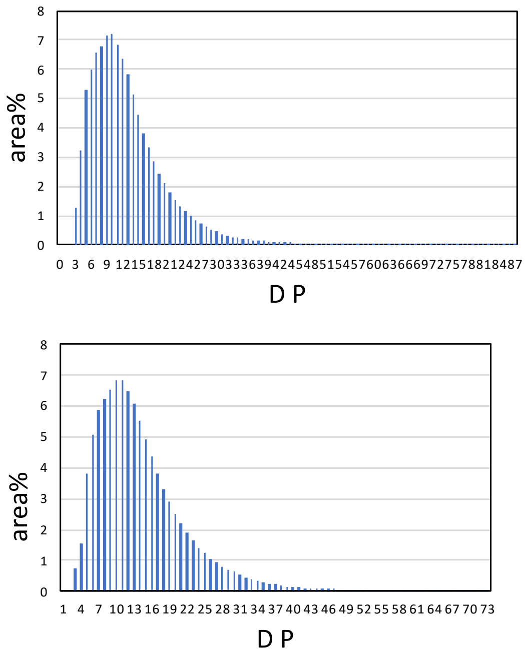

b *ΔCMI294C*

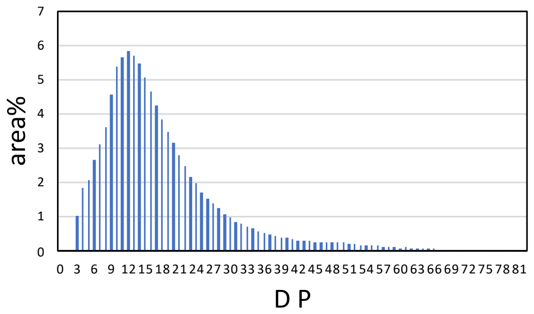

c *ΔCMS197C*

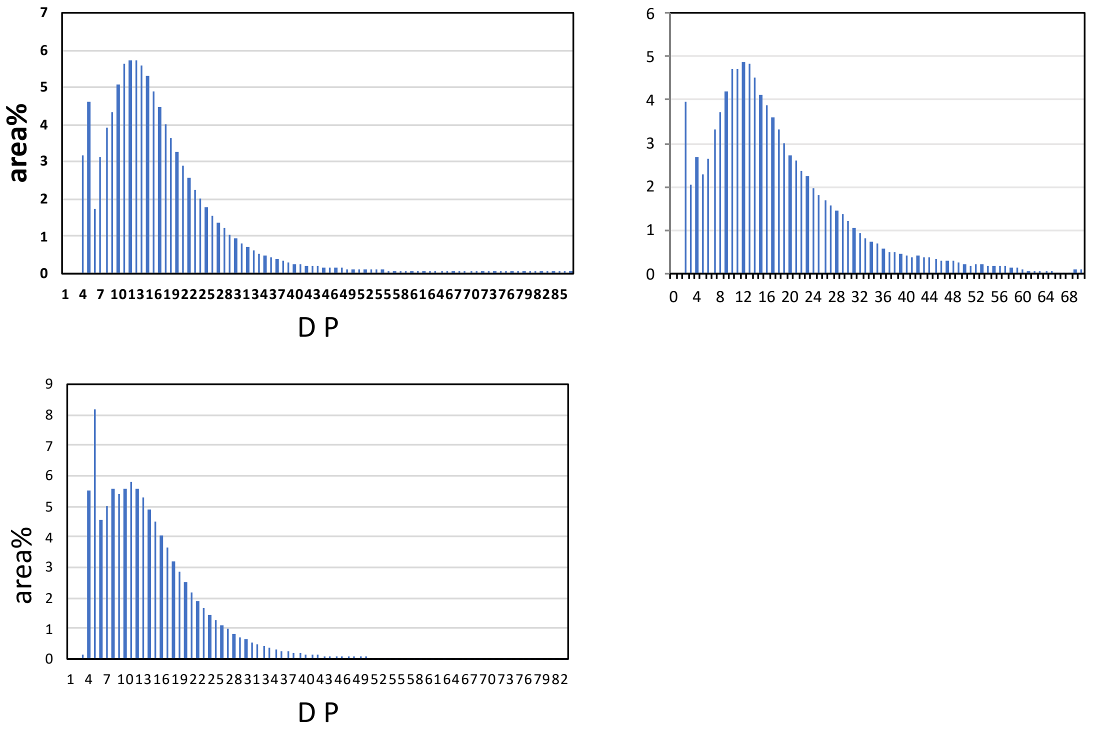

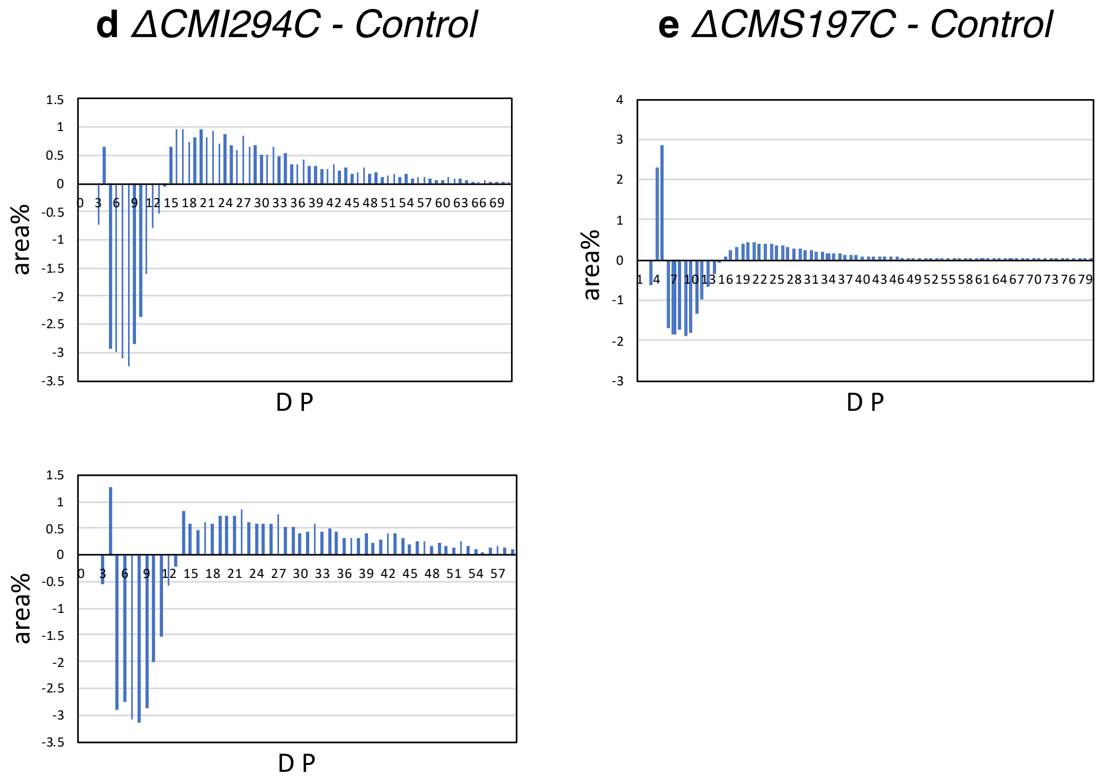

Fig. S2. Supplementary data of Fig. 7. Comparison of the chain-length distribution between starch from the control strain and those from the *ΔCMI294C* and *ΔCMS197C* strains. Starch samples were isolated from late-log phase cells grown under continuous illumination with constant air bubbling containing 2% CO_2. The chain-length profiles (a–c) and differences in chain-length profiles (d, e) are shown. The abundance of chains with individual degrees of polymerization (DP) was plotted as area % of the chromatograms obtained by the chapillary electrophoresis.
